# InBody bioelectrical impedance research in clinical nutrition: a bibliometric analysis of trends, applications, and evidence gaps, 2002–2025

**DOI:** 10.3389/fnut.2026.1896369

**Published:** 2026-09-28

**Authors:** Kun-Wei Tsai, Shih-Wei Yang, Malcolm Koo

**Affiliations:** 1 Division of Geriatrics, Dalin Tzu Chi Hospital, Buddhist Tzu Chi Medical Foundation, Dalin, Chiayi, Taiwan; 2 Department of Education, National Chiayi University, Minsyong, Chiayi, Taiwan; 3 Department of Styling and Cosmetology, Tainan University of Technology, Tainan City, Taiwan; 4 Department of Medical Research, Dalin Tzu Chi Hospital, Buddhist Tzu Chi Medical Foundation, Dalin, Chiayi, Taiwan; 5 Dalla Lana School of Public Health, University of Toronto, Toronto, ON, Canada

**Keywords:** bibliometric analysis, bioelectrical impedance analysis, body composition, clinical nutrition, DSM-MFBIA, InBody, phase angle, sarcopenia

## Abstract

**Background:**

InBody analyzers are widely used for body composition assessment in clinical nutrition research, but no platform-specific bibliometric study has mapped the development, dominant topics, or evidence gaps of the InBody literature. This study examined InBody-related research from 2002 to 2025.

**Methods:**

English-language original research articles were retrieved from the Science Citation Index Expanded within the Web of Science (WoS) Core Collection using the search term “InBody.” Records were analyzed using Bibliometrix and VOSviewer. Analyses included descriptive bibliometrics, country and institutional productivity, journal productivity, citation impact, author-keyword co-occurrence, trend-topic analysis, and logistic growth modeling. Cross-database validation was conducted using Scopus and PubMed.

**Results:**

The final WoS corpus included 406 articles across 217 sources, with an annual growth rate of 19.7% and a mean of 17.7 citations per article; 294 articles (72.4%) were published from 2020 onward. South Korea contributed the largest fractional share (14.6%), while the United States produced the highest mean annual citation rate among the top 10 countries (4.93 citations/year). The logistic model fitted cumulative output closely (R^2^ = 0.97), with an estimated saturation ceiling of approximately 1,172 articles and a projected peak annual output of about 75 articles around 2028. The most-cited articles centered on validation against dual-energy X-ray absorptiometry (DXA). Author-keyword mapping identified seven clusters, including a clinically oriented cluster linking distinct but related themes of sarcopenia, older adults, phase angle, hemodialysis, malnutrition, and frailty. Recent trend topics included phase angle, non-alcoholic fatty liver disease, frailty, young adults, and weight loss. Cross-database validation showed broadly consistent temporal, geographic, and source patterns across WoS, Scopus, and PubMed; all 12 leading WoS author keywords also appeared in Scopus, of which nine ranked within the Scopus top 10.

**Conclusion:**

InBody-related research is expanding rapidly and shifting from device validation toward clinical application, particularly in geriatric and clinical nutrition. Three priorities emerge: (i) device-model-specific and population-specific validation, particularly under-represented regions; (ii) population- and condition-specific phase-angle reference ranges; and (iii) longitudinal and intervention-responsive designs rather than additional cross-sectional DXA comparisons. Addressing these priorities may support more consistent, evidence-based interpretation of InBody-derived body composition measures in nutrition and clinical practice.

## 1 Introduction

Body composition analysis provides a structured assessment of fat mass, skeletal muscle mass, bone mass, and body water compartments (1). These measures support the assessment and management of obesity, sarcopenia, and malnutrition and are used to monitor dietary, exercise, and pharmacological interventions (2, 3). They also inform metabolic health evaluation, cardiovascular risk stratification, and physical performance assessment (4–6). In clinical nutrition, accurate measurement of body composition can help identify patients at nutritional risk, guide nutritional support, and evaluate changes during treatment.

Several technologies are available for body composition assessment, each with distinct strengths and practical constraints. Dual-energy X-ray absorptiometry (DXA) is widely regarded as a reference method for measuring bone mineral density, fat mass, and lean mass, but its cost, radiation exposure, and requirement for fixed equipment limit routine use in many clinical and field settings. Skinfold measurements are portable and inexpensive but depend strongly on examiner technique. Hydrostatic weighing and air displacement plethysmography assess body volume through densitometric principles but require specialized equipment. Bioelectrical impedance analysis (BIA) offers a more practical alternative because it is noninvasive, rapid, and relatively inexpensive. However, BIA measurements are influenced by factors such as hydration status, recent physical activity, food intake, and electrode placement (7, 8).

An additional limitation is that BIA does not represent a single standardized measurement technology. Devices differ in measurement frequency, electrode configuration, body segmentation, posture, signal processing, and proprietary prediction algorithms, and these differences can affect the resulting estimates of body composition (9). Consequently, validation findings obtained with one BIA system cannot necessarily be generalized to another. This device dependence provides a rationale for examining the evidence base of individual commercial platforms rather than treating all BIA instruments as interchangeable.

InBody analyzers (InBody Co., Ltd., Seoul, South Korea) represent a commercially deployed implementation of multifrequency segmental BIA. Founded in 1996 by Dr. Kichul Cha, the company introduced its first commercial device in 1998 with the aim of addressing limitations of conventional BIA approaches that depend on population-derived empirical regression equations based on age, sex, and ethnicity (10, 11). InBody devices use direct segmental multi-frequency bioelectrical impedance analysis (DSM-MFBIA), in which impedance is measured directly and independently across five body segments (right arm, left arm, trunk, right leg, and left leg) at multiple frequencies rather than inferred from a single whole-body measurement. This segmental configuration is intended to reduce reliance on single whole-body estimates and to improve the assessment of regional lean mass and body water distribution (12). Nevertheless, InBody outputs remain dependent on device-specific hardware and proprietary algorithms, and individual models may differ in measurement frequency, electrode configuration, posture, and access to raw impedance parameters (9). These differences make model-specific validation important for clinical interpretation.

The validity of InBody-derived measurements has been examined against DXA and other reference methods in several populations. At the group level, studies have reported acceptable agreement for measures such as total lean mass and fat mass in healthy adults, although systematic biases vary by device model, sex, age, hydration status, and clinical condition. McLester et al. (13) compared three InBody models with DXA and found that the devices could provide practical alternatives when DXA was unavailable, while also identifying model-specific differences in estimates of fat mass and fat-free mass. Other studies have similarly shown that measurement performance varies across populations and clinical settings (14–17). These findings indicate that evidence derived from one model or population should be applied cautiously to other InBody devices or patient groups.

The distinction between platform-specific and device-agnostic evidence is particularly relevant because the broader BIA literature is large and methodologically heterogeneous. A recent bibliometric analysis identified 9,471 BIA-related publications over 20 years, with nutrition and dietetics as the dominant research domain and sarcopenia and phase angle as emerging topics (18). That analysis mapped BIA as a broad field encompassing multiple technologies and manufacturers. It therefore could not determine how the evidence surrounding a single commercial platform has developed, which applications have received the greatest attention, or whether important model-, population-, and disease-specific gaps remain.

A platform-specific bibliometric analysis of InBody research can address these questions at a level closer to clinical use. By limiting the analysis to one BIA platform, some of the technological heterogeneity inherent in device-agnostic analyses can be reduced, allowing closer examination of validation studies, clinical applications, thematic development, and evidence gaps relevant to specific models and populations. Accordingly, this study aimed to map the development of the InBody research literature, characterize its major contributors and thematic trends, and identify areas requiring further investigation, with particular attention to nutritional assessment, geriatric medicine, and chronic disease management.

## 2 Materials and methods

### 2.1 Data source and search strategy

A bibliometric analysis was conducted using the Science Citation Index Expanded (SCI-Expanded) database within the Web of Science (WoS) Core Collection (Clarivate Analytics, Philadelphia, PA, USA). The search was performed on May 3, 2026 using the Topic (TS) field, which includes titles, abstracts, author keywords, and KeyWords Plus, with the exact search term “InBody.” No additional Boolean operators or controlled vocabulary were applied because the brand-specific term was expected to identify publications referring to the commercial bioimpedance platform. The search was not restricted by publication year at initial retrieval but was subsequently limited to articles published from 2002 to 2025. Only English-language original research articles were included in the final analysis (Figure 1).

**Figure 1:**
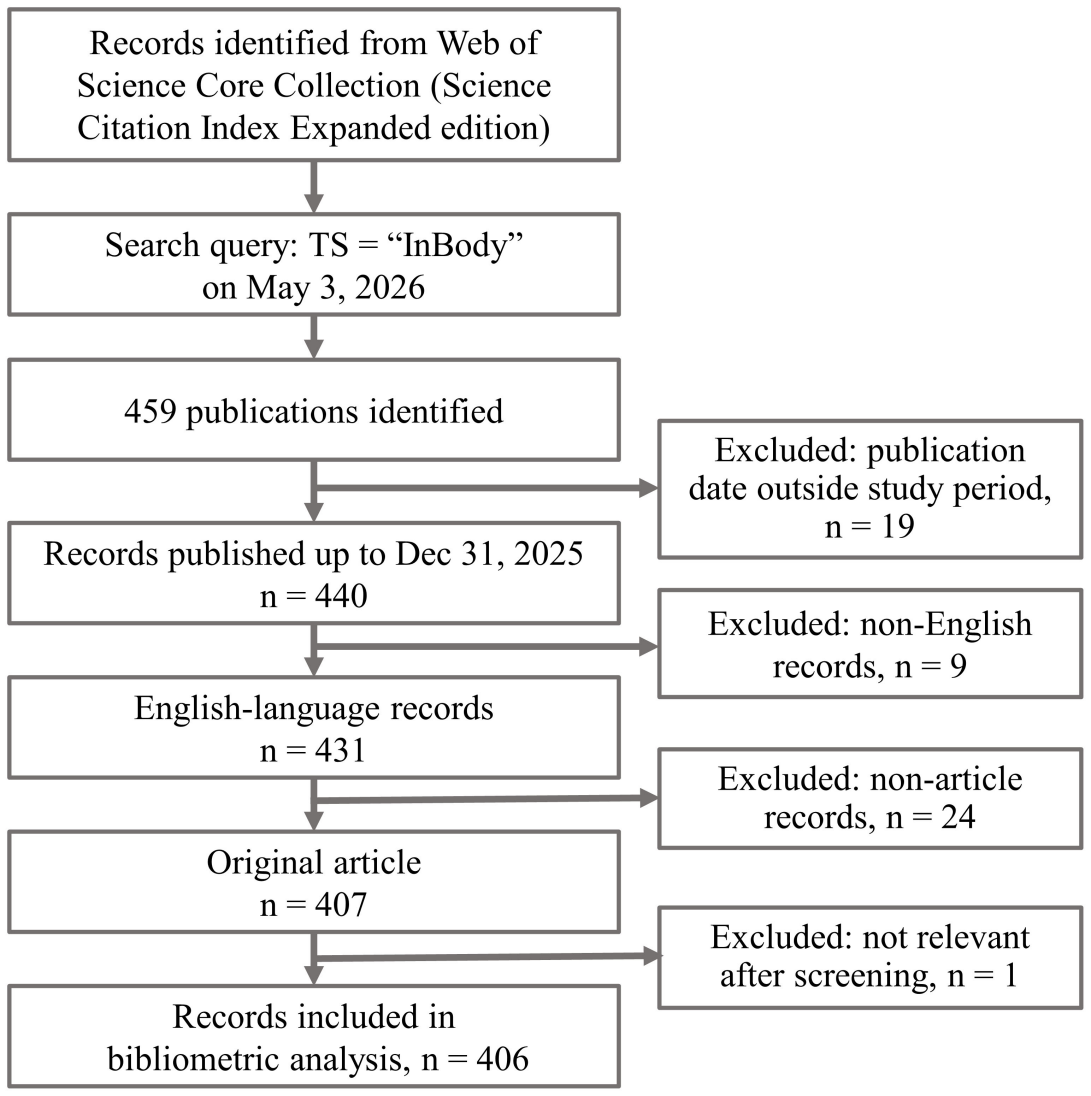
Study flow diagram.

Because the WoS Topic field includes KeyWords Plus in addition to author-visible fields, a sensitivity search was performed using only titles, abstracts, and author keywords: TI = “InBody” OR AB = “InBody” OR AK = “InBody.” The same year, language, document-type, and database restrictions were applied. Records retrieved from the Topic-field search and the author-visible-field sensitivity search were compared at the record level, and discordant records were manually reviewed for relevance to InBody analyzers. An expanded search incorporating variant expressions, including “In-Body,” “InBody analyzer,” “InBody analyser,” “InBody device,” and DSM-MFBIA, returned the same record set, supporting the comprehensiveness of “InBody” as a standalone search term within SCI-Expanded. One false-positive record was excluded because “InBody” appeared as an author surname in a parenthetical citation rather than as a reference to the measurement device.

### 2.2 Bibliometric analysis

Retrieved records were exported in plain-text format and analyzed using R version 4.2.0 and Bibliometrix version 5.0 (Naples, Italy) (19). The Biblioshiny web interface was used for generating trend topic analysis. VOSviewer version 1.6.20 was used for author-keyword co-occurrence mapping (20).

Descriptive bibliometric indicators were calculated including annual publication output, annual growth rate, number of sources, average citations per document, average document age, mean co-authors per document, and proportion of internationally co-authored publications. Annualized citation impact was calculated as total WoS citation count divided by years elapsed since publication up to 2026.

#### 2.2.1 Country and regional level productivity

Country- and region-level productivity was analyzed using a customized R script rather than the default Bibliometrix extraction routine to preserve country and regional labels as recorded in WoS. Country attribution was based on the corresponding-author affiliation extracted from the reprint author field of each WoS record. Affiliation addresses were cross-checked using region-specific patterns, including city names, institutional names, and regional labels, to address publication-level indexing inconsistencies. When multiple corresponding-author countries or regions were listed, fractional counting was applied so that each article contributed a total weight of 1.0, divided equally among the contributing countries or regions. The complete R script, including pattern lists and disambiguation logic, is provided in Supplementary File S1.

#### 2.2.2 Institutional productivity

Institutional productivity was analyzed using a fractional counting approach applied to the normalized university affiliation field (AU_UN) extracted by Bibliometrix from the WoS C1 affiliation field, which records the institutional affiliations of all contributing authors. For each paper, fractional credit of 1/N was assigned to each of the N distinct institutions listed, such that total credit per paper summed to 1.0. Institution names were normalized by converting to uppercase, collapsing internal whitespace, and removing non-alphanumeric characters.

This approach differs from the default Bibliometrix biblioAnalysis() output, which counts raw author-affiliation row occurrences. Because an article with multiple authors from the same institution can generate multiple affiliation rows, the default count reflects author-institution appearances rather than article-level institutional contribution and may inflate institutional output when several co-authors share the same affiliation. In the present corpus, the mean number of distinct institutions per article was 2.39, indicating that raw affiliation-row counts would substantially exceed fractional article-level counts. Two citation impact indicators were calculated: citations per article and mean annual citations per article. The latter was used as the preferred metric for cross-institutional comparison because it accounts for differences in publication age. Institutions with fewer than three fractional articles were excluded from the institutional impact analysis.

#### 2.2.3 Journal productivity and citation impact analysis

Journals were ranked by number of included articles. For each journal, total citation count, 2024 Journal Impact Factor (JIF), WoS category, and JIF quartile were recorded. For journals without a current JIF owing to delisting, the most recent available indexing information was reported. The Normalized Global Citation Score (NGCS) for individual articles was calculated using Bibliometrix, dividing total citation count by the expected citation rate for documents published in the same year, thereby adjusting for the citation accumulation advantage of older publications.

#### 2.2.4 Keyword harmonization and co-occurrence network analysis

A custom thesaurus comprising 192 entries, including 134 merge rules and 58 generic-term exclusions, was applied during VOSviewer term extraction. Merge rules addressed three categories of redundancy: (1) hyphenation and punctuation variants (e.g., “body-composition” and “body composition”); (2) acronyms and their expanded forms (e.g., “BIA,” “bioimpedance,” and “bioelectrical impedance analysis” consolidated to the latter); and (3) lexical synonyms (e.g., “lean body mass” merged with “fat-free mass”). Methodologically distinct concepts were kept separate. Generic terms reflecting study design rather than substantive concepts (e.g., “risk,” “prevalence,” “association”) were excluded to reduce noise. The thesaurus is provided in Supplementary File S2.

Author keyword co-occurrence network analysis was conducted using VOSviewer. Among 976 unique author keywords, 40 met the minimum occurrence threshold of five publications. Co-occurrence strengths were calculated using fractional counting and normalized using association strength. Network clustering used the VOSviewer modularity-based algorithm with a resolution parameter of 1.0, with 10 random starts, 10 iterations per run, and a random seed of 0. To evaluate the sensitivity of the cluster structure to the resolution parameter, the analysis was repeated at resolution values of 0.75, 1.25, and 1.50 while holding the keyword set, normalization method, counting method, random starts, iterations, and random seed constant. The resolution 1.0 solution was retained as the primary analysis, and cluster membership was compared across all four solutions.

#### 2.2.5 Trend-topic analysis

Trend-topic analysis was conducted in Biblioshiny using harmonized author keywords. Keywords were plotted on a temporal axis according to the median publication year of the articles in which they appeared, with horizontal bars representing the interquartile range. The analysis used a minimum keyword frequency of five and a maximum of five keywords per year.

#### 2.2.6 Life-cycle analysis

A logistic growth model was fitted to the cumulative annual publication data using the life cycle function implemented in Biblioshiny: P(t) = K / (1 + exp(–b(t – t0))), where P(t) is the cumulative number of publications at time t, K is the saturation ceiling, b is the growth rate parameter, and t0 is the inflection point. Three key model parameters were estimated: the saturation ceiling (K); the peak year (T_*m*_), at which annual output is projected to be highest; and the growth duration (Δt), defined as the estimated time from 10% to 90% of K. Model fit was assessed using R^2^ and root mean square error (RMSE).

#### 2.2.7 Multi-database validation analysis

To assess the database-dependence of the WoS-based findings, a cross-database validation analysis was conducted using Scopus and PubMed. These validation datasets were analyzed separately rather than merged with the WoS corpus because WoS, Scopus, and PubMed differ in indexing scope, metadata structure, citation coverage, and field definitions. The validation was designed to examine whether the main bibliometric patterns observed in WoS were broadly reproduced in independent bibliographic sources, rather than to establish record-for-record agreement across databases.

For Scopus, the validation search used author-visible fields to reduce retrieval from trade-name and manufacturer metadata. The final Scopus query was:

(TITLE({InBody}) OR ABS({InBody}) OR AUTHKEY({InBody})) AND PUBYEAR > 2001 AND PUBYEAR < 2026 AND (LIMIT-TO(DOCTYPE, “ar”)) AND (LIMIT-TO(LANGUAGE, “English”)).

This search retrieved 456 records. After exclusion of one false-positive surname record, the final Scopus validation dataset included 455 records. For comparison, the broader Scopus TITLE-ABS-KEY({InBody}) search retrieved 1,807 records, with field diagnostics showing substantial retrieval through KEY({InBody}), TRADENAME({InBody}), and MANUFACTURER({InBody}). The restricted Scopus search was selected to exclude trade-name and manufacturer indexing fields, which are not analogous to the WoS Topic field; therefore, the Scopus validation deliberately compares like-for-like author-visible content rather than maximal retrieval.

For PubMed, the validation search captured records in which InBody was explicitly mentioned in the Title/Abstract or Other Term fields:

(“InBody”[Title/Abstract] OR “InBody”[Other Term]) AND (“2002/01/01”[Date - Publication]: “2025/12/31”[Date - Publication]) AND english[Language] AND “journal article”[Publication Type] NOT (review[Publication Type] OR systematic review[Publication Type] OR meta-analysis[Publication Type] OR editorial[Publication Type] OR letter[Publication Type] OR comment[Publication Type] OR news[Publication Type]).

Records retrieved from Scopus and PubMed were screened using eligibility criteria aligned with the primary WoS corpus. Records were excluded if InBody did not refer to the InBody analyzer, device, or platform, or if the publication was not an original research article.

The validation analysis compared the WoS, Scopus, and PubMed datasets descriptively in terms of annual publication trends, leading corresponding-author countries or regions, and leading publication sources. For country and regional comparison, database-reported corresponding-author outputs from Bibliometrix were used. This supplementary comparison was separate from the main WoS country/region analysis, which used customized fractional counting. Countries or regions were included if they appeared within the top-10 rank positions in at least one database. Ranks were assigned within each database according to article counts; countries or regions with the same count shared the same rank, and the next distinct count received the next consecutive rank. Percentages were not reported because the three databases had different denominators. No *post hoc* geopolitical harmonization was applied; therefore, these comparisons were interpreted as descriptive cross-database checks rather than fully standardized national or regional productivity estimates.

As a supplementary check of cross-database geographic consistency, Kendall's τ-b rank correlations were calculated for country/region article counts across the WoS, Scopus, and PubMed datasets. All countries/regions appearing in at least one database-specific country/region output were included, and countries/regions absent from a given database were assigned an article count of zero. Kendall's τ-b was selected because it accounts for ties in ranked data.

For publication-source comparison, sources were ranked by publication count within each database. Sources with the same publication count shared the same rank, and the next distinct count received the next consecutive rank. All sources tied within the top-five rank positions in at least one database were retained.

Author-keyword profiles were compared between WoS and Scopus, with the same synonym-merging and stop-word rules applied to both outputs. PubMed was excluded from this comparison because its keyword metadata are less consistently structured for bibliometric ranking. Within each database, author keywords were ranked by occurrence count with tied counts sharing a rank; as a result, the WoS top-10 rank positions encompassed 12 keywords. Each of these WoS-leading keywords was then located in the Scopus output, with its Scopus occurrence count and rank reported when it appeared within the Scopus top-10, or labeled “Outside top 10” if present in Scopus but at a lower rank.

## 3 Results

### 3.1 Search sensitivity, overview, and publication growth trends

The primary WoS Topic-field search retrieved 407 records after applying year, language, and document-type restrictions. Manual review excluded one false-positive record, an HIV/SHIV (human immunodeficiency virus and simian–human immunodeficiency virus) virology study in which “Inbody” appeared only as an author surname in a cited reference within the abstract, yielding 406 eligible articles for analysis. The author-visible-field sensitivity search retrieved 404 records. Record-level comparison showed that all 404 records were also retrieved by the Topic-field search, including the same false-positive surname record. After exclusion of this false-positive record, the author-visible-field search would have yielded 403 eligible articles. The three additional records retrieved only through the Topic-field search contained “InBody 720” in KeyWords Plus but not in the title, abstract, or author keywords, and all three were manually confirmed as relevant to InBody analyzers. These findings indicate that the Topic-field strategy provided marginally greater sensitivity without reducing search precision.

The final WoS corpus comprised 406 articles published between 2002 and 2025 across 217 sources, with an annual growth rate of 19.7% (Figure 2A). The overall corpus had an average of 17.7 citations per document, 6.8 co-authors per document, and an average document age of 5.2 years, with 26.1% of documents involving international co-authorships.

**Figure 2:**
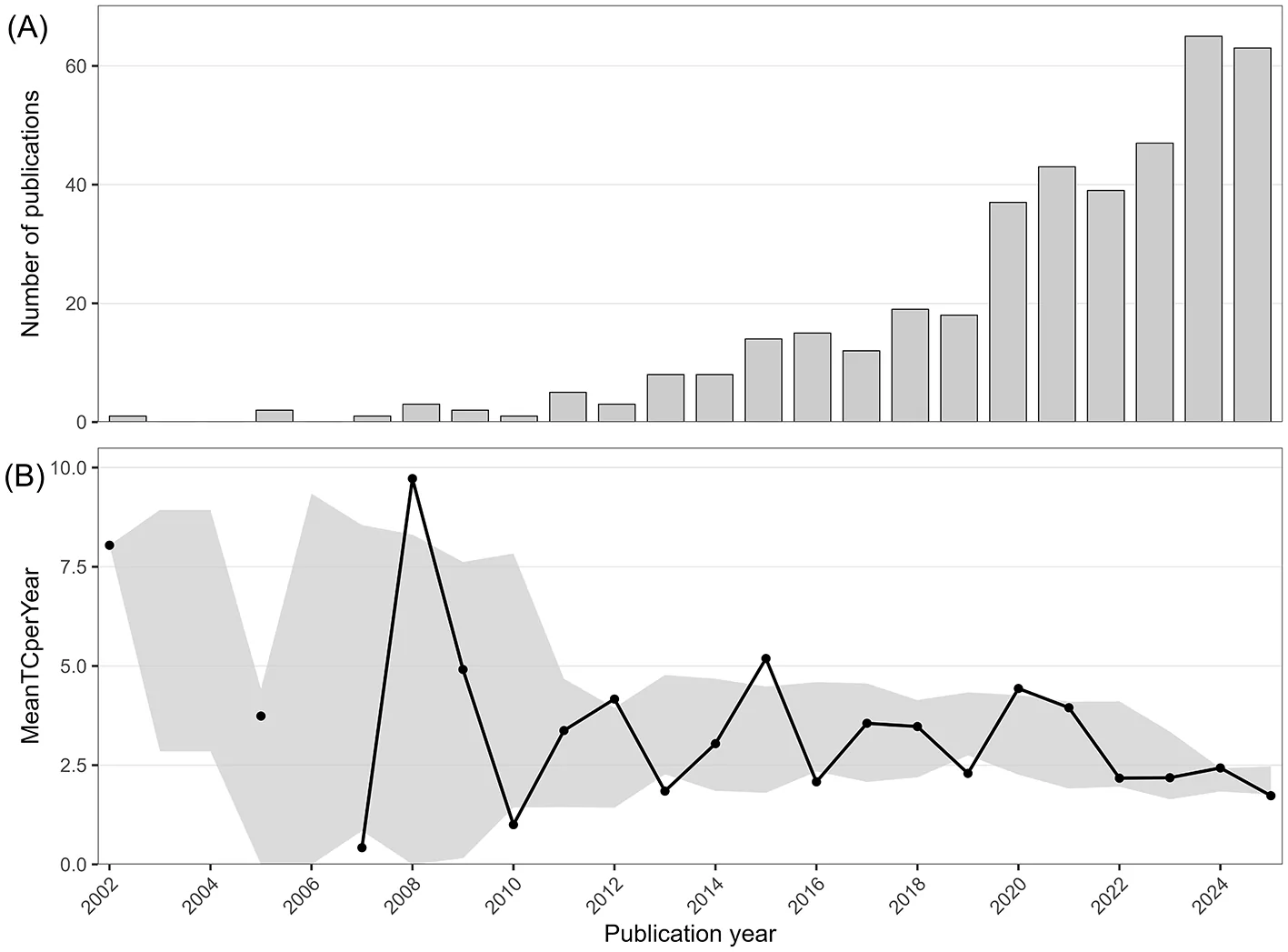
Annual publication output and annualized citation impact of InBody publications, 2002–2025. Panel **(A)** shows the number of publications by year. Panel **(B)** shows the mean citation rate per year (MeanTCperYear), calculated for each article as the Web of Science Core Collection citation count divided by (2026 – publication year + 1). The shaded area represents the 95% confidence interval of the yearly mean.

Publication output was sparse during the first decade of the search window. Only 18 articles were published between 2002 and 2012, with no eligible publications in 2003, 2004, or 2006. A gradual increase became apparent from 2013 onward, with 94 articles published between 2013 and 2019, including early acceleration in 2015 (*n* = 14) and 2016 (*n* = 15). A marked acceleration occurred from 2020 onward. The period from 2020 to 2025 accounted for 294 publications, representing 72.4% of the WoS corpus. Annual output increased from 37 articles in 2020 to a peak of 65 in 2024, followed by 63 articles in 2025.

The logistic growth model showed excellent fit to the cumulative annual publication data (R^2^ = 0.97, RMSE = 3.58). The model estimated a saturation ceiling of approximately 1,172 publications, a projected peak annual output of 75 publications per year around 2028, and a growth duration of 17.2 years. As of 2025, the 406 publications in the WoS corpus represented 34.6% of the estimated saturation ceiling, placing the InBody literature in the accelerating phase of the projected growth curve.

Citation impact, expressed as mean total citations per year, showed a different temporal pattern (Figure 2B). Early publications had relatively high per-year citation rates, with the 2008 cohort recording the highest value (MeanTCperYear = 9.72). From 2011 onward, mean annual citation rates stabilized, with most years falling in the range of approximately 2–4 citations per year per document. The most recent cohorts from 2023 to 2025 showed lower mean annual citation rates, consistent with their shorter citation accumulation time.

### 3.2 Country and regional productivity

The geographic distribution of publications showed concentration among a small number of countries and regions (Table 1). South Korea was the most productive country, contributing 59.3 fractional articles, representing 14.6% of the WoS corpus, but had a comparatively modest mean annual citation rate of 2.49. China ranked second with 45.5 fractional articles (11.2%), followed by the United States with 37.3 fractional articles (9.2%) and Japan with 35.0 fractional articles (8.6%). Despite ranking third by output, the United States recorded the highest mean annual citation rate (4.93) and the highest total citation count (1,314), indicating proportionally greater citation impact per publication.

**Table 1:** Top 10 countries and regions contributing to InBody-related research, 2002 to 2025.

Rank	Country/Region	Total articles (%)	MCP %	Total citations	Mean annual citations/Article
1	South Korea	59.3 (14.6)	2.2	995.0	2.49
2	China	45.5 (11.2)	12.1	472.2	2.27
3	United States	37.3 (9.2)	0.9	1,314.0	4.93
4	Japan	35.0 (8.6)	2.9	708.5	2.85
5	Poland	17.5 (4.3)	2.9	166.5	1.96
6	Iran	14.2 (3.5)	8.2	100.5	2.24
7	Czech Republic	13.5 (3.3)	3.7	139.5	1.18
8	Brazil	12.3 (3.0)	2.7	229.7	2.44
9	Taiwan	11.5 (2.8)	4.3	185.0	2.58
10	Hungary	11.0 (2.7)	0	77.0	2.41

MCP, multiple-country publication. Article counts reflect fractional counting. For articles with multiple corresponding authors from different countries, credit was distributed equally across contributing countries.

The remaining six countries each contributed fewer than 18 fractional articles. Hungary recorded no multiple-country publications, whereas China showed the highest multiple-country publication rate at 12.1%, followed by Iran at 8.2%. The top three countries accounted for 35.0% of total fractional publication output, and the top 10 collectively accounted for 63.3% of the WoS corpus.

### 3.3 Institutional productivity

Table 2 presents the top institutions ranked by fractional article count and by mean annual citations per article. By fractional article count, the National and Kapodistrian University of Athens ranked first with 8.0 fractional articles. Comenius University Bratislava and Sichuan University each contributed 6.5 fractional articles. Seoul National University ranked seventh by output, with 5.2 fractional articles, but second by citation impact, with 5.58 mean annual citations per article.

**Table 2:** Top 10 institutions contributing to InBody-related research ranked by fractional article count (left) and mean annual citations per article (right), 2002–2025.

Rank	Institution, Country	Fractional articles	Mean annual citation/Article	Rank	Institution, Country	Fractional articles	Mean annual citation/Article
1	National & Kapodistrian University of Athens, Greece	8.0	0.84	1	University of Southern California, USA	3.0	6.34
2	Comenius University Bratislava, Slovakia	6.5	4.69	2	Seoul National University, South Korea	5.2	5.58
3	Sichuan University, China	6.5	2.49	3	Comenius University Bratislava, Slovakia	6.5	4.69
4	Palacký University Olomouc, Czech Republic	5.8	2.33	4	Sichuan University, China	6.5	2.49
5	University of Ljubljana, Slovenia	5.4	1.77	5	Palacký University Olomouc, Czech Republic	5.8	2.33
6	University of Belgrade, Serbia	5.3	2.14	6	Medical University Sofia, Bulgaria	4.0	2.19
7	Seoul National University, South Korea	5.2	5.58	7	University of Belgrade, Serbia	5.3	2.14
8	Medical University Sofia, Bulgaria	4.0	2.19	8	Southwest Medical University, China	3.0	2.06
9	Masaryk University, Czech Republic	3.9	0.40	9	King Saud University, Saudi Arabia	3.3	1.79
10	Tehran University of Medical Sciences, Iran	3.8	1.30	10	University of Ljubljana, Slovenia	5.4	1.77

The University of Southern California ranked first by citation impact, with 6.34 mean annual citations per article. This estimate was based on three fractional articles, including two publications from 2008 (21) and 2012 (22) that each accumulated more than 130 citations. The divergence between output-based and citation-based rankings suggests that high publication volume and high citation impact reflect distinct dimensions of institutional contribution to the InBody literature, with several high-volume institutions absent from the top-10 by citation impact and vice versa. Some institutions with high raw author-affiliation counts in the default Bibliometrix output, such as Shanghai Jiao Tong University and Pusan National University, did not appear in the fractional top 10. This difference illustrates how raw author-affiliation counting can overestimate institutional output when several co-authors from the same institution contribute to the same article.

### 3.4 Journal productivity

A total of 12 journals are listed within the top five ranks because of tied publication counts (Table 3). *PLoS ONE* and *Scientific Reports* were tied at rank one, each publishing 12 articles. The *International Journal of Morphology* ranked second with 11 articles and had a 2024 Journal Impact Factor of 0.5 (Q4). Three journals shared rank three with 10 articles each: *Frontiers in Nutrition, International Journal of Environmental Research and Public Health*, and *Medicine*. *Frontiers in Nutrition* had the highest Journal Impact Factor among these three journals (5.1, Q1), whereas the *International Journal of Environmental Research and Public Health* had been delisted from WoS in March 2023. The *European Journal of Clinical Nutrition* ranked fifth with seven articles but accumulated 443 total citations, the highest citation count among the listed journals, reflecting the high citation yield of validation-oriented articles in clinical nutrition journals.

**Table 3:** Top journals contributing to InBody-related research, 2002 to 2025.

Rank	Journal	No. articles	Total citations	2024 JIF, WoS Category [JIFQ]
1	*PLoS ONE*	12	235	2.6, Multidisciplinary Sciences [Q2]
1	*Scientific Reports*	12	153	3.9, Multidisciplinary Sciences [Q1]
2	*International Journal of Morphology*	11	109	0.5, Anatomy & Morphology [Q4]
3	*Frontiers in Nutrition*	10	221	5.1, Nutrition & Dietetics [Q1]
3	*International Journal of Environmental Research and Public Health*	10	130	4.6, Public Health [Q1] in 2021^*^
3	*Medicine*	10	73	1.4, Medicine, General & Internal [Q2]
4	*Nutrients*	9	198	5.0, Nutrition & Dietetics [Q1]
5	*European Journal of Clinical Nutrition*	7	443	3.3, Nutrition & Dietetics [Q2]
5	*Frontiers in Endocrinology*	7	140	4.6, Endocrinology & Metabolism [Q1]
5	*Frontiers in Physiology*	7	68	3.4, Physiology [Q1]
5	*Sensors*	7	58	3.5, Instruments & Instrumentation [Q2]
5	*Blood Pressure Monitoring*	7	23	1.2, Peripheral Vascular Disease [Q4]

JIF, Journal Impact Factor; JIFQ, JIF Quartile; WoS, Web of Science. Q1–Q4, journal-impact-factor quartile ranking within WoS subject category. 12 journals listed due to tied ranks. ^*^International Journal of Environmental Research and Public Health was delisted from the WoS Master Journal List in March 2023.

### 3.5 Most-cited articles

The ten most-cited articles are presented in Table 4. The most-cited article, published in the *Journal of Clinical Densitometry* in 2020, compared the reliability of various InBody body composition analyzers against DXA in healthy men and women (13), accumulating 338 total citations, 48.3 citations per year, and an NGCS of 12.7. The second- and third-ranked articles also focused on body composition assessment using bioimpedance compared with dual-energy X-ray absorptiometry.

**Table 4:** Ten most-cited articles on InBody-related research, 2002 to 2025.

Rank	Title, Publication year (Reference number)	Journal	Total citations	Citations/Year	NGCS
1	Reliability and agreement of various InBody body composition analyzers as compared to dual-energy x-ray absorptiometry in healthy men and women, 2020 (13)	*Journal of Clinical Densitometry*	338	48.3	12.7
2	Assessing body composition with DXA and bioimpedance: Effects of obesity, physical activity, and age, 2008 (35)	*Obesity*	217	11.4	1.2
3	Accuracy of an eight-point tactile-electrode impedance method in the assessment of total body water, 2002 (36)	*European Journal of Clinical Nutrition*	193	7.7	1.0
4	Concordance between muscle mass assessed by bioelectrical impedance analysis and by dual energy X-ray absorptiometry: A cross-sectional study, 2015 (37)	*BMC Musculoskeletal Disorders*	169	14.1	3.0
5	Sarcopenia is a predictor of postoperative respiratory complications in patients with esophageal cancer, 2015 (24)	*Annals of Surgical Oncology*	168	14.0	2.9
6	Ability of new octapolar bioimpedance spectroscopy analyzers to predict 4-component-model percentage body fat in Hispanic, Black, and White adults, 2008 (15)	*American Journal of Clinical Nutrition*	164	8.6	0.9
7	Validity and reliability of body composition analysers in children and adults, 2008 (21)	*British Journal of Nutrition*	144	7.6	0.8
8	Validation study of multi-frequency bioelectrical impedance with dual-energy X-ray absorptiometry among obese patients, 2014 (38)	*Obesity Surgery*	140	10.8	3.8
9	Comparison between dual-energy X-ray absorptiometry and bioelectrical impedance analyses for accuracy in measuring whole body muscle mass and appendicular skeletal muscle mass, 2018 (23)	*Nutrients*	136	15.2	4.9
10	Utility of multifrequency bioelectrical impedance compared with dual-energy X-ray absorptiometry for assessment of total and regional body composition varies between men and women, 2012 (22)	*Nutrition Research*	135	9.0	2.3

NGCS, Normalized Global Citation Score.

Most of the top 10 were methodological studies evaluating bioimpedance-derived estimates against DXA or other reference methods. The ninth-ranked article, published in *Nutrients* in 2018 (23), recorded 15.2 citations per year and the second-highest Normalized Global Citation Score despite a lower total citation count, indicating disproportionate impact after adjustment for publication year. One highly cited article examined the prognostic relevance of sarcopenia in a surgical oncology population (24), reflecting the clinical application of InBody-derived measurements beyond measurement validation alone.

### 3.6 Author-keyword co-occurrence network analysis

The author-keyword co-occurrence network comprised seven distinct clusters (Figure 3). Cluster 1 (red) represented clinical nutrition, geriatric, and chronic-disease applications, anchored by body composition, sarcopenia, older adults, phase angle, hemodialysis, malnutrition, frailty, body water, and weight loss. These terms represent clinically distinct constructs that were grouped according to their keyword co-occurrence structure; their placement in the same cluster should therefore be interpreted as bibliometric connectivity rather than clinical equivalence. Cluster 2 (green) captured an anthropometric and population-based theme encompassing obesity, body mass index, children, adolescents, adiposity, anthropometry, lean mass, and overweight. Cluster 3 (blue-gray) reflected a body composition measurement theme including body fat, fat mass, fat-free mass, muscle mass, total body water, bone mineral density, women, and young adults.

**Figure 3:**
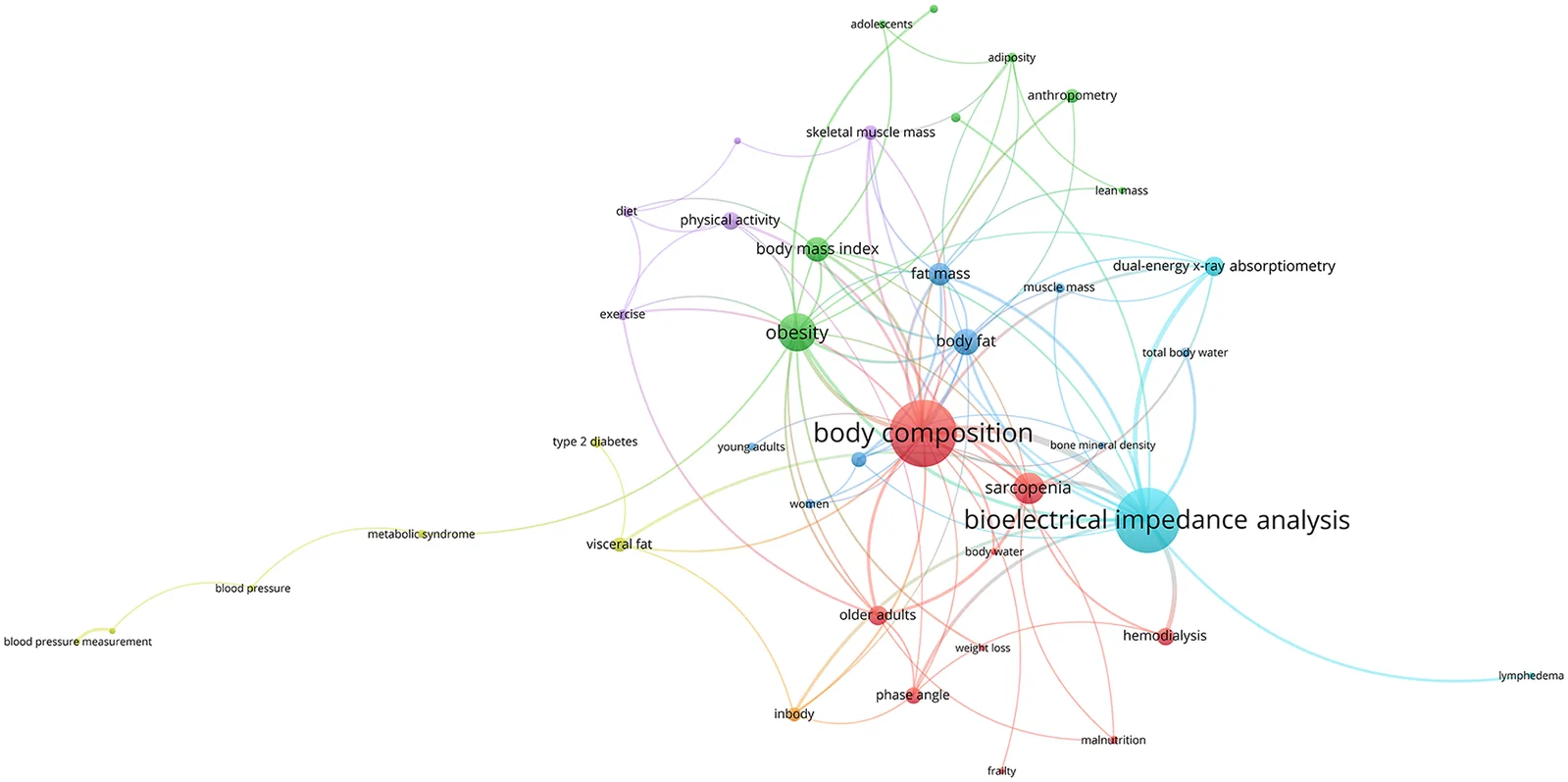
Author-keyword co-occurrence network of InBody-related research, 2002–2025. Node size is proportional to keyword frequency. Edge thickness is proportional to co-occurrence strength. Seven clusters from the primary resolution 1.0 solution are indicated by color.

Cluster 4 (yellow-green) grouped cardiometabolic risk-related keywords including visceral fat, metabolic syndrome, type 2 diabetes, and blood pressure. Cluster 5 (purple) captured a lifestyle and physical health theme comprising physical activity, skeletal muscle mass, exercise, diet, and non-alcoholic fatty liver disease (NAFLD). Cluster 6 (light blue) represented a validation and instrumentation theme centered on bioelectrical impedance analysis, dual-energy X-ray absorptiometry, and lymphedema. Cluster 7 (orange) contained a single device-name term (“InBody”) representing self-referential platform tagging and was not interpreted as a substantive thematic cluster.

Resolution-parameter sensitivity analysis yielded 5, 7, 7, and 10 clusters at resolution values of 0.75, 1.00, 1.25, and 1.50, respectively. Cluster assignments were identical at resolutions 1.00 and 1.25. The principal clinical-nutrition grouping was generally stable across the tested resolutions. Sarcopenia, malnutrition, older adults, frailty, hemodialysis, and phase angle remained grouped together at all four resolutions. At resolution 1.50, body composition, body water, and weight loss separated into an additional cluster.

### 3.7 Trend-topic analysis

The trend-topic analysis identified 20 author keywords with sufficient frequency for temporal mapping (Figure 4). Keywords with the earliest median publication years reflected foundational measurement themes: dual-energy X-ray absorptiometry (frequency = 20; median = 2018), total body water (frequency = 8; median = 2019), fat-free mass (frequency = 15; median = 2019), and bioelectrical impedance analysis (frequency = 90; median = 2020). A second group with median years between 2021 and 2023 captured consolidating themes: obesity, fat mass, sarcopenia, body mass index, hemodialysis, body composition, older adults, and skeletal muscle mass, with body composition being the most frequent keyword (frequency = 94; median = 2023).

**Figure 4:**
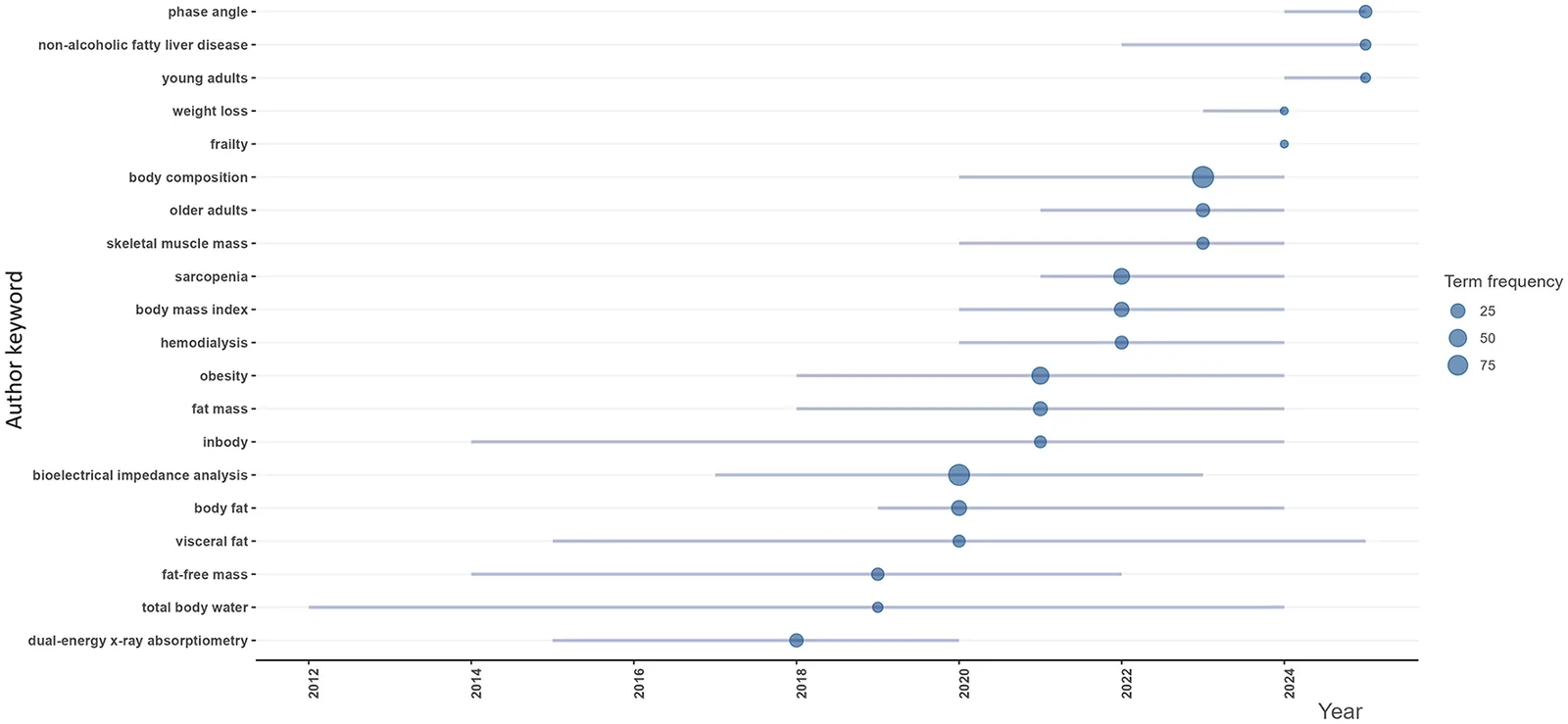
Trend-topic analysis of author keywords in InBody-related research, 2002–2025. Each node represents a keyword plotted at its median publication year, with horizontal bars spanning the interquartile range. Node size is proportional to keyword frequency.

The most recent research directions included phase angle (frequency = 16; median = 2025), non-alcoholic fatty liver disease (median = 2025), young adults (median = 2025), frailty (median = 2024), and weight loss (median = 2024). These emerging topics suggest expanding interest in impedance-derived parameters and metabolic health applications beyond traditional body composition estimation.

### 3.8 Cross-database validation of WoS-derived patterns

The cross-database validation analysis included independent Scopus and PubMed searches in addition to the primary WoS corpus (Table 5). The Scopus search retrieved 456 records; after exclusion of one false-positive surname record, 455 records were retained in the final Scopus validation dataset. The PubMed search retrieved 673 records; nine records were excluded, including eight records assigned to publication year 2026 and one false-positive surname record, leaving 664 records in the final PubMed validation dataset.

**Table 5:** Search strategies, retrieval results, and validation datasets across Web of Science, Scopus, and PubMed.

Database	Search field	Search strategy	Filters applied	Records retrieved, n	Records excluded after screening	Final validation dataset, n	Main role in validation
Web of Science Core Collection	Topic field (TS); sensitivity search in title, abstract, and author keywords (TI/AB/AK)	TS = “InBody”; sensitivity search using TI/AB/AK = “InBody”	2002–2025; English; original research articles; SCI-Expanded	407	1 false-positive surname record	406	Primary bibliometric corpus
Scopus	TITLE, ABS, AUTHKEY	(TITLE({InBody}) OR ABS({InBody}) OR AUTHKEY({InBody})) AND PUBYEAR > 2001 AND PUBYEAR < 2026 AND (LIMIT-TO(DOCTYPE, “ar”)) AND (LIMIT-TO(LANGUAGE, “English”))	2002–2025; English; article	456	1 false-positive surname record	455	Independent bibliographic validation; annual publication, source, country/region, and author-keyword comparison
PubMed	Title/Abstract; Other Term	(“InBody”[Title/Abstract] OR “InBody”[Other Term]) AND (“2002/01/01”[Date - Publication]: “2025/12/31”[Date - Publication]) AND english[Language] AND “journal article”[Publication Type] NOT (review[Publication Type] OR systematic review[Publication Type] OR meta-analysis[Publication Type] OR editorial[Publication Type] OR letter[Publication Type] OR comment[Publication Type] OR news[Publication Type])	2002–2025; English; journal article; non-review publication types	673	9 records: 8 records assigned to publication year 2026; 1 false-positive surname record	664	Biomedical coverage validation; annual publication, source, and country/region comparison

SCI-Expanded, Science Citation Index Expanded; TITLE, ABS, and AUTHKEY refer to Scopus title, abstract, and author-keyword fields, respectively.

The annual publication trajectories in Scopus and PubMed broadly paralleled the WoS corpus (Figure 5). All three databases showed limited output before 2013, gradual expansion during 2013–2019, and marked acceleration after 2020. Absolute counts differed across databases, with PubMed retrieving more publications than WoS and Scopus in recent years, particularly during 2020–2025. Nevertheless, the same overall temporal pattern was observed across all three sources, supporting the interpretation that InBody-related research has entered a rapid growth phase.

**Figure 5:**
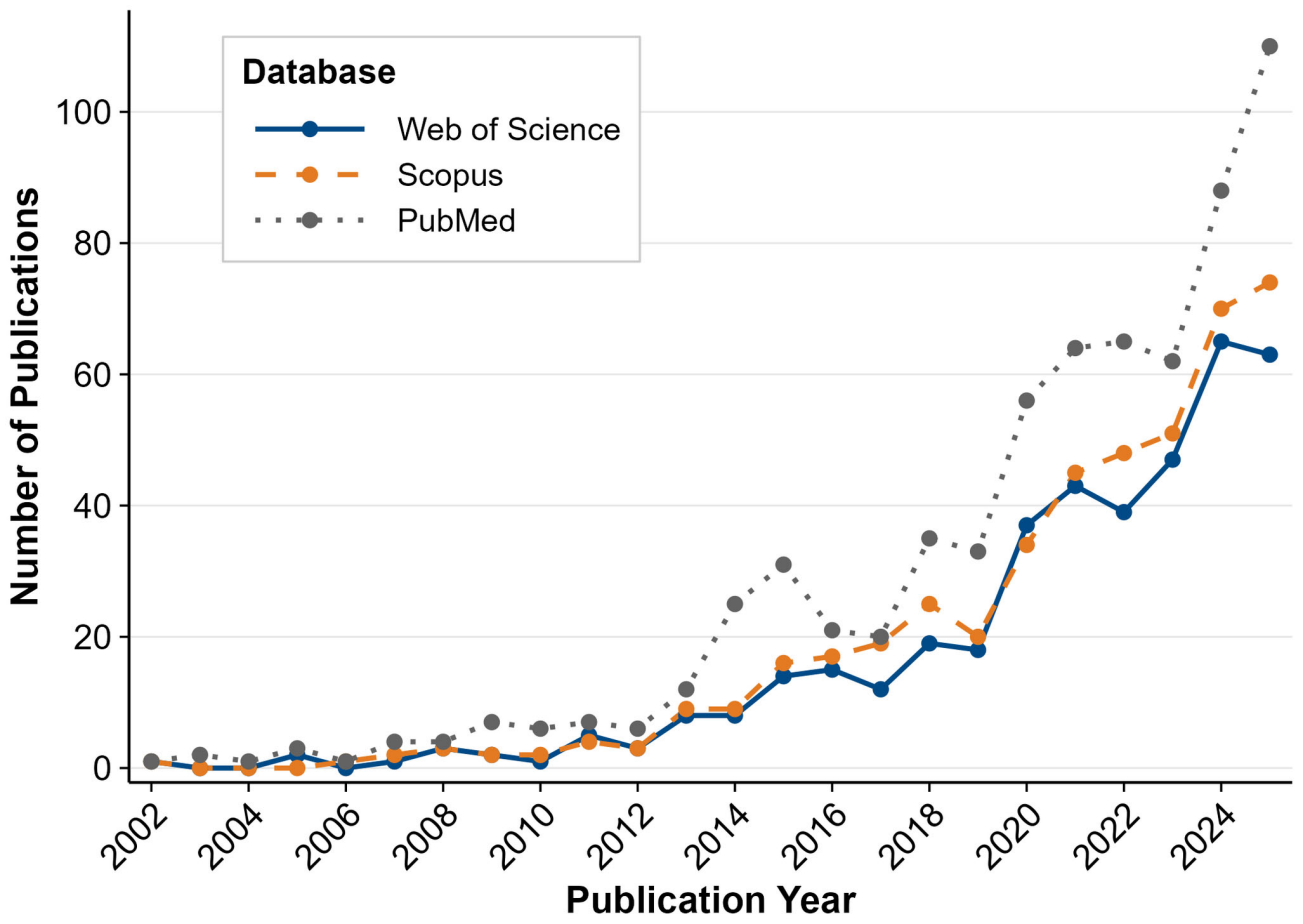
Annual publication counts of InBody-related research across Web of Science, Scopus, and PubMed, 2002–2025.

In the supplementary affiliation-based country and regional validation analysis, the leading corresponding-author countries and regions showed substantial consistency across WoS, Scopus, and PubMed (Supplementary Table S1). This supplementary comparison used raw database-reported corresponding-author counts and therefore differs from the customized fractional country/region analysis presented in Table 1. Nine countries or regions appeared within the top-10 rank positions in all three databases: China, South Korea, the United States, Japan, Poland, Czech Republic, Iran, Brazil, and Hungary. China ranked first in WoS and Scopus and third in PubMed, whereas Japan ranked fourth in WoS, third in Scopus, and first in PubMed. South Korea ranked second across all three databases, and the United States ranked third in WoS and fourth in both Scopus and PubMed. Slovakia and Serbia appeared within the top-10 rank positions in WoS and Scopus but not in PubMed, whereas Australia and Portugal appeared only within the PubMed top-10 rank positions. These findings indicate that the geographic pattern observed in WoS was broadly consistent with the patterns observed in Scopus and PubMed, although the comparison should be interpreted descriptively because no *post hoc* geopolitical harmonization was applied.

Across 62 countries/regions, Kendall's τ-b showed significant positive correlations between WoS and Scopus (τ-b = 0.669, *p* < 0.001), WoS and PubMed (τ-b = 0.667, *p* < 0.001), and Scopus and PubMed (τ-b = 0.517, *p* < 0.001). These findings indicate that the geographic distribution of InBody-related publications was broadly consistent across databases, although database coverage, metadata structure, and country/region attribution methods should be considered when interpreting rank-order differences.

Leading publication sources also showed considerable overlap across databases (Supplementary Table S2). *PLoS ONE* and *Scientific Reports* ranked first in WoS, whereas *PLoS ONE* was the leading source in Scopus and the *Annual International Conference of the IEEE Engineering in Medicine and Biology Society* was the leading source in PubMed. Overall, of the 16 sources ranked within the top-five source positions in at least one database, 13 appeared in at least two database-specific source lists. Most leading WoS sources also appeared in Scopus and/or PubMed, including *PLoS ONE, Scientific Reports, Frontiers in Nutrition, International Journal of Environmental Research and Public Health, Medicine, Nutrients, Sensors*, and *Blood Pressure Monitoring*. Three sources were specific to a single validation dataset: *Journal of Physical Education and Sport* in Scopus, and the *Annual International Conference of the IEEE Engineering in Medicine and Biology Society* and *Journal of Artificial Organs* in PubMed.

The leading author-keyword profile was also largely consistent between WoS and Scopus (Supplementary Table S3). Because tied ranks were retained, the WoS top-10 rank positions included 12 author keywords. All 12 WoS-derived leading keywords were identified in the Scopus validation dataset. Nine appeared within the Scopus top-10 rank positions, including the three highest-ranked keywords in both databases: body composition, bioelectrical impedance analysis, and obesity. The remaining three WoS-derived keywords, dual-energy x-ray absorptiometry, hemodialysis, and phase angle, were present in Scopus but ranked outside the top-10 rank positions. Supplementary Figure S1 further showed a Scopus trend-topic pattern compatible with the WoS-based trend-topic analysis, with a shift from measurement-oriented terms toward clinical and population-specific applications, including sarcopenia, older adults, phase angle, and metabolic conditions. Overall, the validation analyses suggest that the main temporal, geographic, and publication-source patterns observed in WoS were broadly reproduced in Scopus and PubMed, while the leading author-keyword profile was strongly reproduced in Scopus.

## 4 Discussion

This bibliometric analysis of 406 InBody-related publications from 2002 through 2025 shows a field undergoing rapid structural transformation, moving from a narrow device-validation literature toward a broader clinical research landscape involving nutritional assessment, chronic disease management, and geriatric health. Four patterns are especially notable: dissociation between publication volume and citation impact; a thematic shift from instrumentation to clinical application; a coherent network linking geriatric, nutritional, and chronic-disease applications; and geographic concentration that leaves important evidence gaps.

### 4.1 Volume without proportional impact

A central cross-cutting observation is the dissociation between publication volume and citation impact at multiple levels of analysis. South Korea led in article output (14.6%) but recorded a lower mean citation rate than several other top-10 countries (2.49 citations/year), whereas the United States produced proportionally fewer articles but generated nearly twice the citation impact per publication (4.93 citations/year). This pattern was also evident at the institutional level: institutions dominating article counts under raw Bibliometrix counting did not appear among the sources of the ten most-cited articles, which originated predominantly from European and North American research groups.

At the journal level, the *International Journal of Morphology* ranked second by article count but had a JIF of 0.5 (Q4), whereas the *European Journal of Clinical Nutrition* ranked fifth and accumulated 443 total citations from only seven articles, the highest citation yield of any listed journal. These patterns suggest that high-volume producers within the InBody literature are not necessarily the same entities driving citation impact. Device availability may facilitate frequent publications, but the field's clinical credibility will depend more on independent validation, longitudinal outcome studies, and intervention trials than on further increases in raw publication volume.

### 4.2 Comparison with review-level evidence on validation and clinical utility

The bibliometric patterns identified here are broadly consistent with findings from systematic and narrative reviews of the wider BIA literature. A systematic review in surgical and oncological populations reported substantial variability among BIA prediction equations and poorer performance when equations developed in healthy populations were applied to patients with altered body composition, supporting the need for population-specific validation (16). Similarly, a systematic review of multifrequency BIA in adults with obesity found generally high correlations with reference methods at the group level but lower individual-level agreement, with accuracy improving when obesity-specific algorithms were used (25). These findings are consistent with the continued influence of validation studies in our analysis and support a shift from generic device–reference comparisons toward population- and model-specific validation.

Device heterogeneity further limits extrapolation across BIA platforms. A recent narrative review emphasized differences among BIA instruments in measurement frequency, electrode configuration, access to raw impedance parameters, and proprietary algorithms, all of which can affect clinical interpretation (9). This supports the platform-specific approach used in the present study, as validation findings from one BIA device or model may not apply to another.

Review-level evidence also provides context for the emerging clinical themes identified in our analysis. A 2024 systematic review and meta-analysis of 18 studies reported moderate-to-good diagnostic performance of phase angle for sarcopenia screening, although performance varied by ethnicity, body mass index, health status, and diagnostic criteria, suggesting that population- and condition-specific reference values remain necessary (26). A 2023 systematic review and meta-analysis of 12 randomized trials involving 2,420 dialysis patients found lower mortality and better blood-pressure control with bioimpedance-guided volume management, but no clear benefit for hospitalization, major cardiovascular events, or left-ventricular mass index (27). These findings support the growing clinical interest in BIA identified in our analysis, while indicating that its utility varies across settings and outcomes. Most review-level evidence, however, evaluates BIA or multifrequency BIA as a broad category rather than InBody devices specifically, which limits direct extrapolation to individual InBody models.

### 4.3 From measurement equivalence to clinical utility

Most of the ten most-cited articles addressed, in some form, the comparative accuracy of BIA against DXA or another reference method. This convergence is understandable because establishing measurement validity is a necessary precondition for clinical adoption. It is also limiting because the bibliometric evidence suggests that the field has remained anchored to validation studies longer than the platform's clinical diffusion might warrant. The lag between thematic emergence (median publication years 2022–2025 for clinical themes) and citation accumulation likely accounts in part for the apparent dissociation between top-cited content (validation-focused) and recent topical structure (clinically focused).

The normalized citation analysis reinforces this point. The ninth-ranked article, which examined DXA-BIA agreement specifically for appendicular skeletal muscle mass, recorded the second-highest NGCS (4.9) despite its lower raw citation count, pointing to disproportionate impact per publication in clinically targeted validation work. Together with the trend-topic analysis showing skeletal muscle mass and sarcopenia reaching median publication years of 2023 and 2022, respectively, this pattern suggests that the field is beginning to shift its validation agenda toward clinically meaningful body composition compartments. Future validation efforts should prioritize population-specific cutoff derivation and longitudinal tracking of muscle mass changes in response to nutritional and exercise interventions.

### 4.4 Geriatric and clinical nutrition applications

The co-occurrence network and trend topic analysis together indicate a densely connected bibliometric domain spanning clinical nutrition, geriatric, and chronic-disease applications. Within this cluster, sarcopenia, older adults, phase angle, hemodialysis, malnutrition, and frailty co-occurred despite representing distinct clinical constructs. In particular, sarcopenia and malnutrition should not be interpreted as equivalent conditions; their co-clustering indicates that these topics are frequently studied within overlapping clinical research contexts. The sensitivity analysis supported this interpretation because sarcopenia and malnutrition remained in the same algorithmically generated cluster across resolution parameters from 0.75 to 1.50. This connectivity is clinically plausible because populations with chronic disease often require rapid and reproducible assessment of muscle mass, fluid status, and nutritional risk, and InBody's segmental architecture makes such assessment practically feasible across these settings.

Phase angle is a clinically relevant emerging topic in the InBody literature. Its recent median publication year (2025), together with its increasing prominence in the keyword network, points to expanding interest in this parameter as a complement to conventional body composition estimates. Unlike fat mass or skeletal muscle mass, which are derived through prediction equations applied to raw impedance data, phase angle is less dependent on proprietary body-composition prediction equations than derived estimates such as fat mass or skeletal muscle mass. This equation-independent derivation may make phase angle particularly useful in populations for whom standard InBody prediction outputs are less valid, although clinical interpretation still requires age-, sex-, body mass index-, and ethnicity-specific reference values (28). Physiologically, phase angle reflects cellular health, membrane integrity, and fluid distribution, which may explain its growing clinical role in nutritional assessment. Lower phase angle values have been associated with sarcopenia, supporting its potential as a practical screening marker when DXA is unavailable (29). Its emergence in both the broader BIA literature (18) and the InBody-specific corpus analyzed in this study suggests that future research should prioritize population- and disease-specific phase angle reference ranges for InBody devices before routine clinical implementation.

The hemodialysis subtheme within this clinical cluster warrants separate comment. Its co-occurrence with sarcopenia and malnutrition reflects the nutritional vulnerability of patients with end-stage renal disease, in whom fluid overload, muscle wasting, and protein-energy malnutrition coexist and are difficult to disentangle using conventional anthropometry (30). InBody's extracellular-to-total body water ratio output provides a direct index of fluid distribution that is clinically actionable for nutritional monitoring and fluid management, a capability that distinguishes it meaningfully from single-frequency BIA devices (31). Despite this clinical relevance, hemodialysis research within the corpus showed moderate normalized citation impact, pointing to a domain where research activity has not yet translated into the outcome evidence that would support guideline-level nutritional recommendations for InBody use in renal nutrition practice.

### 4.5 Emerging research directions

The trend topic analysis identifies three emerging themes: NAFLD, young adults, and frailty. Their recent median publication years and narrow interquartile ranges indicate concentrated, recent activity that has not yet accumulated sufficient citation mass to establish evidentiary credibility.

NAFLD, now increasingly referred to as metabolic dysfunction-associated steatotic liver disease (MASLD), is particularly relevant from a nutritional standpoint. Visceral fat accumulation, estimable via InBody, is strongly associated with hepatic steatosis and disease progression, and body composition assessment may have a useful role in NAFLD risk stratification and monitoring response to dietary or lifestyle intervention (32). The co-occurrence network placed NAFLD within the lifestyle and physical activity cluster rather than the cardiometabolic cluster, suggesting that hepatic applications of InBody remain structurally peripheral to the field's core concerns despite the global prevalence of metabolic liver disease.

The young-adult topic reflects the broadening of InBody research beyond geriatric clinical management into preventive, screening, and public health applications. Frailty, in contrast, points back to geriatric nutrition and functional decline. Better phenotyping will be needed if InBody-derived parameters are to support clinically meaningful frailty classification in older adults.

### 4.6 Geographic concentration and equity gaps

The top 10 countries account for 63.3% of the corpus, with the near-complete absence of sub-Saharan African, South Asian, and Latin American research output beyond Brazil. This geographic concentration is not simply a reflection of research capacity disparities; it may also reflect device access inequity. InBody analyzers carry procurement and maintenance costs that may limit availability in low- and middle-income health systems, precisely where the dual burden of undernutrition and obesity-related disease is most acute and where accurate, portable body composition assessment would have the greatest public health value.

The normative and validation data underpinning InBody's predictive outputs are derived largely from East Asian, European, and North American populations. Applying these outputs to populations with different body composition distributions may introduce systematic measurement bias that remains difficult to quantify because the relevant validation research has not yet been conducted. Addressing this gap requires expanding device access and prioritizing population-specific validation studies as a condition of responsible clinical deployment.

### 4.7 A prioritized research agenda for clinical nutrition practice

Drawing on the evidence gaps identified across all result domains, three priorities stand out as clinically most consequential for clinical nutrition practice.

First, device-model-specific validation across clinical populations should be the field's most immediate priority. The InBody product range includes multiple models differing in electrode configuration and frequency, yet most validation studies in this corpus do not specify the device model used. A clinician selecting or interpreting an InBody output needs to know whether the validation evidence applies to the specific device in their setting. This requires systematic model-specific studies across diverse clinical populations, stratified by sex, adiposity, and clinical condition.

Second, establishing population-specific phase angle reference ranges using InBody devices is an important near-term priority. Phase angle has emerged as one of the more clinically promising InBody-derived parameters, particularly for sarcopenia screening and nutritional assessment in older adults and hemodialysis patients. Because phase angle is comparatively independent of prediction equations, it offers a more transparent bridge between measurement and clinical interpretation, but it currently lacks standardized normative data that would support routine clinical implementation.

Third, longitudinal responsiveness of impedance-derived parameters deserves prioritization over further cross-sectional DXA comparisons. The most-cited literature has repeatedly addressed group-level agreement between InBody and DXA in non-clinical adults. What is now needed is evidence that InBody-derived estimates, or raw parameters such as phase angle, can detect clinically meaningful change over time in patients undergoing nutritional intervention, disease management, or rehabilitation. Future bibliometric analyses should also examine whether emerging modalities such as wearable bioimpedance sensors have developed sufficiently distinct publication patterns to warrant platform-specific mapping (33).

### 4.8 Cross-database validation and database-dependence of the findings

The multi-database validation analysis provides additional support for the main WoS-based findings while also showing the importance of interpreting bibliometric results in relation to database coverage. The Scopus and PubMed validation datasets showed publication trajectories that were broadly consistent with the WoS corpus, with sparse output before 2013, gradual expansion during 2013–2019, and marked acceleration from 2020 onward. This agreement suggests that the observed growth of InBody-related research is unlikely to be an artifact of WoS indexing alone. However, the higher number of PubMed records in recent years also indicates that absolute publication counts are database-dependent and should not be interpreted as fixed estimates of the total literature. The larger PubMed retrieval likely reflects broader biomedical-journal indexing, including non-SCIE-listed clinical journals, and the inclusion of MeSH-derived “Other Term” matches that have no exact analog in WoS metadata.

The cross-database country/region and publication-source comparisons further support the pattern-level consistency of the main bibliometric findings, but they should be interpreted descriptively. The supplementary country/region comparison was based on Biblioshiny-derived country/region outputs generated from the available author-affiliation metadata in each database. This approach differs from the main WoS country/region analysis, which used customized fractional counting based on corresponding-author affiliations. In particular, PubMed does not consistently provide a standardized corresponding-author affiliation field; therefore, PubMed-derived country/region outputs should be interpreted as affiliation-based descriptive indicators rather than strict corresponding-author productivity estimates. Nevertheless, country-level affiliation assignment is generally less sensitive to author-position differences than institution-level assignment (34), and supplementary Kendall's τ-b analyses showed significant positive rank correlations across all database pairs when all countries/regions were considered. Differences in rank order across these analyses may therefore reflect database coverage, metadata structure, and counting method rather than true disagreement. Similarly, the publication-source comparison showed substantial recurrence of journals and conference sources across databases, although this recurrence does not imply identical source rankings. These findings support the use of WoS as the primary bibliometric corpus while indicating that the major temporal, geographic, and publication-source patterns were broadly reproduced in Scopus and PubMed.

The author-keyword validation between WoS and Scopus shows that the main topical profile was not specific to the WoS metadata structure. All leading WoS-derived author keywords appeared in Scopus, and most were also within the Scopus top-10 rank positions. The strongest recurrent terms, including body composition, bioelectrical impedance analysis, obesity, sarcopenia, body fat, and body mass index, indicate that the core intellectual structure of this literature is centered on body composition assessment and its clinical applications. Taken together, the validation findings strengthen the interpretation that InBody-related research has evolved from a measurement-focused literature toward a broader clinical and population-health research field.

### 4.9 Limitations

This study has several limitations inherent to bibliometric methodology. First, the main analysis was restricted to English-language original research articles indexed in WoS, and relevant studies in other languages or outside WoS may have been missed. Second, the search strategy was based on the brand-specific term “InBody”; therefore, the findings characterize InBody-related research rather than the broader literature on bioelectrical impedance analysis or body composition assessment. Third, although Scopus and PubMed were used for cross-database validation, the three databases differ in indexing scope, metadata structure, citation coverage, and field definitions. Accordingly, the validation analyses should be interpreted as pattern-level comparisons rather than exact record-for-record replication. Fourth, bibliometric indicators measure publication and citation patterns rather than study quality; citation influence should therefore not be interpreted as evidence of methodological validity or clinical reliability. Fifth, keyword analyses depend on author keyword selection. Although thesaurus harmonization was applied, residual terminology variation may have affected the co-occurrence clusters and trend-topic results. Finally, logistic growth projections assume relatively stable publication conditions and may not account for changes in clinical guidelines, replacement of InBody by competing technologies, or shifts in research funding priorities.

## 5 Conclusions

This bibliometric analysis of 406 WoS-indexed InBody-related articles published from 2002 through 2025 points out a field in a rapid growth phase, with 72.4% of publications appearing from 2020 onward. The most-cited literature remains concentrated in cross-sectional validation against dual-energy X-ray absorptiometry, while recent thematic trends show increasing attention to clinical nutrition, geriatric medicine, phase angle, frailty, sarcopenia, and metabolic health. Independent cross-database validation in Scopus and PubMed produced broadly consistent temporal, geographic, source, and keyword patterns, supporting the stability of the main findings beyond WoS indexing alone. Three priorities emerge for strengthening clinical utility: (i) device-model-specific and population-specific validation, with particular attention to under-represented regions; (ii) population- and condition-specific phase-angle reference ranges; and (iii) longitudinal and intervention-responsive designs rather than additional cross-sectional DXA comparisons. Progress in these areas may support more consistent, evidence-based interpretation of InBody-derived body composition measures in nutrition and clinical practice.

## Data Availability

The bibliographic records analyzed in this study were retrieved from the Web of Science Core Collection and Scopus under institutional subscription licenses and from PubMed, which is publicly accessible. Web of Science and Scopus records are available from their respective providers subject to licensing restrictions. The R scripts and VOSviewer thesaurus are provided in the Supplementary material. Further inquiries can be directed to Malcolm Koo, m.koo@utoronto.ca.

## References

[1] Schoeller DA. Bioelectrical impedance analysis. What does it measure? Ann N Y Acad Sci. (2000) 904:159–62. doi: 10.1111/j.1749-6632.2000.tb06441.x 10865730

[2] Ceniccola GD Castro MG Piovacari SMF Horie LM Corrêa FG Barrere APN . Current technologies in body composition assessment: advantages and disadvantages. Nutrition. (2019) 62:25–31. doi: 10.1016/j.nut.2018.11.028 30826596

[3] Moroni A Gasparri C Perna S Rondanelli M Micheletti Cremasco M. Appendicular Skeletal Muscle Mass (ASMM) and Fat-Free Mass (FFM) DXA-BIA estimations for the early identification of sarcopenia/low muscle mass in middle-aged women. Nutrients. (2024) 16:3897. doi: 10.3390/nu16223897 39599682 PMC11597462

[4] Oh CM Bang JI Lee SY Lee JK Chai JW Oh SW. An analysis of age-related body composition changes and metabolic patterns in Korean adults using FDG-PET/CT health screening data. Diabetes Metab J. (2025) 49:92–104. doi: 10.4093/dmj.2024.0057 39219438 PMC11788554

[5] Nikoohemmat M Ramezani Ahmadi A Valizadeh A Moteshakereh SM Yari-Boroujeni R Seifi Z . Association between body composition indices and vascular health: a systematic review and meta-analysis. Eat Weight Disord. (2025) 30:3. doi: 10.1007/s40519-025-01714-7 39799535 PMC11725544

[6] Moon JR. Body composition in athletes and sports nutrition: an examination of the bioimpedance analysis technique. Eur J Clin Nutr. (2013) 67:S54–9. doi: 10.1038/ejcn.2012.165 23299872

[7] Fosbøl MØ Zerahn B. Contemporary methods of body composition measurement. Clin Physiol Funct Imaging. (2015) 35:81–97. doi: 10.1111/cpf.12152 24735332

[8] Kyle UG Bosaeus I De Lorenzo AD Deurenberg P Elia M Gómez JM . Bioelectrical impedance analysis–part I: review of principles and methods. Clin Nutr. (2004) 23:1226–43. doi: 10.1016/j.clnu.2004.06.004 15380917

[9] Dupertuis YM Jimaja W Beardsley Levoy C Genton L. Bioelectrical impedance analysis instruments: how do they differ, what do we need for clinical assessment? Curr Opin Clin Nutr Metab Care. (2025) 28:379–87. doi: 10.1097/MCO.0000000000001142 40667712 PMC12337901

[10] Cha K. History of InBody. Seoul: InBody Co., Ltd. (2020). InBody White Paper.

[11] InBody. InBody Technology. InBody (n.d.). Available online at: https://inbodyusa.com/general/technology/ (Accessed May 21, 2026).

[12] Ling CHY de Craen AJM Slagboom PE Gunn DA Stokkel MPM Westendorp RGJ . Accuracy of direct segmental multi-frequency bioimpedance analysis in the assessment of total body and segmental body composition in middle-aged adult population. Clin Nutr. (2011) 30:610–5. doi: 10.1016/j.clnu.2011.04.001 21555168

[13] McLester CN Nickerson BS Kliszczewicz BM McLester JR. Reliability and agreement of various InBody body composition analyzers as compared to dual-energy X-ray absorptiometry in healthy men and women. J Clin Densitom. (2020) 23:443–50. doi: 10.1016/j.jocd.2018.10.008 30472111

[14] Larsen MN Krustrup P Araújo Póvoas SC Castagna C. Accuracy and reliability of the InBody 270 multi-frequency body composition analyser in 10-12-year-old children. PLoS ONE. (2021) 16:e0247362. doi: 10.1371/journal.pone.0247362 33770106 PMC7996997

[15] Gibson AL Holmes JC Desautels RL Edmonds LB Nuudi L. Ability of new octapolar bioimpedance spectroscopy analyzers to predict 4-component-model percentage body fat in Hispanic, Black, and White adults. Am J Clin Nutr. (2008) 87:332–8. doi: 10.1093/ajcn/87.2.332 18258622

[16] Haverkort EB Reijven PLM Binnekade JM de van der Schueren Earthman CP Gouma DJ . Bioelectrical impedance analysis to estimate body composition in surgical and oncological patients: a systematic review. Eur J Clin Nutr. (2015) 69:3–13. doi: 10.1038/ejcn.2014.203 25271012

[17] Lewis JI Friis H Mupere E Wells JCK Grenov B. Calibration of bioelectrical impedance analysis against deuterium dilution for body composition assessment in stunted Ugandan children. J Nutr. (2023) 153:426–34. doi: 10.1016/j.tjnut.2022.12.028 36894235

[18] Niu C Zhang P Zhang C Dong J Liang H Xiao D . Evolution of research trends and emerging hotspots in bioelectrical impedance analysis over the last two decades: a bibliometric analysis. J Int Soc Sports Nutr. (2025) 22:2523381. doi: 10.1080/15502783.2025.2523381 40569570 PMC12203698

[19] Aria M Cuccurullo C. *bibliometrix*: an R-tool for comprehensive science mapping analysis. J Informetr. (2017) 11:959–75. doi: 10.1016/j.joi.2017.08.007

[20] van Eck NJ Waltman L. Software survey: VOSviewer, a computer program for bibliometric mapping. Scientometrics. (2010) 84:523–38. doi: 10.1007/s11192-009-0146-3 20585380 PMC2883932

[21] Jensky-Squires NE Dieli-Conwright CM Rossuello A Erceg DN McCauley S Schroeder ET. Validity and reliability of body composition analysers in children and adults. Br J Nutr. (2008) 100:859–65. doi: 10.1017/S0007114508925460 18346304

[22] Anderson LJ Erceg DN Schroeder ET. Utility of multifrequency bioelectrical impedance compared with dual-energy X-ray absorptiometry for assessment of total and regional body composition varies between men and women. Nutr Res. (2012) 32:479–85. doi: 10.1016/j.nutres.2012.05.009 22901555

[23] Lee SY Ahn S Kim YJ Ji MJ Kim KM Choi SH . Comparison between dual-energy X-ray absorptiometry and bioelectrical impedance analyses for accuracy in measuring whole body muscle mass and appendicular skeletal muscle mass. Nutrients. (2018) 10:738. doi: 10.3390/nu10060738 29880741 PMC6024648

[24] Ida S Watanabe M Yoshida N Baba Y Umezaki N Harada K . Sarcopenia is a predictor of postoperative respiratory complications in patients with esophageal cancer. Ann Surg Oncol. (2015) 22:4432–7. doi: 10.1245/s10434-015-4559-3 25862583

[25] Becroft L Ooi G Forsyth A King S Tierney A. Validity of multi-frequency bioelectric impedance methods to measure body composition in obese patients: a systematic review. Int J Obes. (2019) 43:1497–507. doi: 10.1038/s41366-018-0285-9 30568268

[26] Zhang J Wang N Li J Wang Y Xiao Y Sha T. The diagnostic accuracy and cutoff value of phase angle for screening sarcopenia: a systematic review and meta-analysis. J Am Med Dir Assoc. (2024) 25:105283. doi: 10.1016/j.jamda.2024.105283 39326856

[27] Horowitz L Karadjian O Braam B Mavrakanas T Weber C. Bioimpedance-guided monitoring of volume status in patients with kidney disease: a systematic review and meta-analysis. Can J Kidney Health Dis. (2023) 10:20543581231185433. doi: 10.1177/20543581231185433 37457623 PMC10338662

[28] Bellido D García-García C Talluri A Lukaski HC García-Almeida JM. Future lines of research on phase angle: strengths and limitations. Rev Endocr Metab Disord. (2023) 24:563–83. doi: 10.1007/s11154-023-09803-7 37043140 PMC10090740

[29] Wu H Ding P Wu J Yang P Tian Y Zhao Q. Phase angle derived from bioelectrical impedance analysis as a marker for predicting sarcopenia. Front Nutr. (2022) 9:1060224. doi: 10.3389/fnut.2022.1060224 36590205 PMC9798294

[30] Hara H Nakamura Y Hatano M Iwashita T Shimizu T Ogawa T . Protein energy wasting and sarcopenia in dialysis patients. Contrib Nephrol. (2018) 196:243–9. doi: 10.1159/000485729 30041234

[31] Pérez-Morales R Donate-Correa J Martín-Núñez E Pérez-Delgado N Ferri C López-Montes A . Extracellular water/total body water ratio as predictor of mortality in hemodialysis patients. Ren Fail. (2021) 43:821–9. doi: 10.1080/0886022X.2021.1922442 33966595 PMC8118427

[32] Ko YH Wong TC Hsu YY Kuo KL Yang SH. The correlation between body fat, visceral fat, and nonalcoholic fatty liver disease. Metab Syndr Relat Disord. (2017) 15:304–11. doi: 10.1089/met.2017.0001 28481662

[33] Arabsalmani N Ghouchani A Jafarabadi Ashtiani S Zamani M. Exploring bio-impedance sensing for intelligent wearable devices. Bioengineering. (2025) 12:521. doi: 10.3390/bioengineering12050521 40428140 PMC12109311

[34] Yu J Yin C Liu L Jia T. A paper's corresponding affiliation and first affiliation are consistent at the country level in Web of Science. arXiv [Preprint]. (2021). doi: 10.48550/arXiv.2101.09426

[35] Völgyi E Tylavsky FA Lyytikäinen A Suominen H Alén M Cheng S. Assessing body composition with DXA and bioimpedance: effects of obesity, physical activity, and age. Obesity. (2008) 16:700–5. doi: 10.1038/oby.2007.94 18239555

[36] Bedogni G Malavolti M Severi S Poli M Mussi C Fantuzzi AL . Accuracy of an eight-point tactile-electrode impedance method in the assessment of total body water. Eur J Clin Nutr. (2002) 56:1143–8. doi: 10.1038/sj.ejcn.1601466 12428182

[37] Buckinx F Reginster JY Dardenne N Croisiser JL Kaux JF Beaudart C . Concordance between muscle mass assessed by bioelectrical impedance analysis and by dual energy X-ray absorptiometry: a cross-sectional study. BMC Musculoskelet Disord. (2015) 16:60. doi: 10.1186/s12891-015-0510-9 25887598 PMC4369090

[38] Faria SL Faria OP Cardeal MDA Ito MK. Validation study of multi-frequency bioelectrical impedance with dual-energy X-ray absorptiometry among obese patients. Obes Surg. (2014) 24:1476–80. doi: 10.1007/s11695-014-1190-5 24464546

