# Supplementary material for: InBody bioelectrical impedance research in clinical nutrition: a bibliometric analysis of trends, applications, and evidence gaps, 2002–2025
Source: Front Nutr. 2026 Sep 28;13:1896369. doi: 10.3389/fnut.2026.1896369 (PMC13656847; doi:10.3389/fnut.2026.1896369)
Supplement: Supplementary file 1 [file Data_Sheet_1.zip › Supplementary Table S3.docx]

**Supplementary Table S3**. Cross-database recurrence of leading Web of Science author keywords in the Scopus validation dataset, 2002–2025.

| **WoS-derived author keyword** | **Web of Science** | |  | **Scopus** | |  | **Cross-database status** |
| --- | --- | --- | --- | --- | --- | --- | --- |
|  | **n** | **rank** |  | **n** | **rank/status** |  |  |
| body composition | 94 | 1 |  | 146 | 1 |  | Present in Scopus top 10 |
| bioelectrical impedance analysis | 90 | 2 |  | 84 | 2 |  | Present in Scopus top 10 |
| obesity | 47 | 3 |  | 57 | 3 |  | Present in Scopus top 10 |
| sarcopenia | 36 | 4 |  | 33 | 6 |  | Present in Scopus top 10 |
| body fat | 30 | 5 |  | 35 | 4 |  | Present in Scopus top 10 |
| body mass index | 27 | 6 |  | 34 | 5 |  | Present in Scopus top 10 |
| fat mass | 24 | 7 |  | 23 | 8 |  | Present in Scopus top 10 |
| dual-energy x-ray absorptiometry | 20 | 8 |  | 15 | Outside top 10 |  | Present in Scopus, outside top 10 |
| older adults | 20 | 8 |  | 23 | 8 |  | Present in Scopus top 10 |
| hemodialysis | 18 | 9 |  | 13 | Outside top 10 |  | Present in Scopus, outside top 10 |
| physical activity | 18 | 9 |  | 24 | 7 |  | Present in Scopus top 10 |
| phase angle | 16 | 10 |  | 15 | Outside top 10 |  | Present in Scopus, outside top 10 |

WoS, Web of Science.

n refers to the number of author-keyword occurrences in the database-specific most-frequent-author-keyword output. Keywords with the same occurrence count were assigned the same rank. Because tied ranks were retained, the WoS top 10 rank positions included 12 keywords. The table includes all WoS author keywords within these top 10 rank positions. For Scopus, exact ranks are shown only when the corresponding keyword appeared within the Scopus top 10 rank positions; keywords present in Scopus but outside this range are labelled as “Outside top 10.” The same synonym-merging and stop-word rules were applied to the WoS and Scopus author-keyword outputs.
