# Supplementary material for: InBody bioelectrical impedance research in clinical nutrition: a bibliometric analysis of trends, applications, and evidence gaps, 2002–2025
Source: Front Nutr. 2026 Sep 28;13:1896369. doi: 10.3389/fnut.2026.1896369 (PMC13656847; doi:10.3389/fnut.2026.1896369)
Supplement: Supplementary file 1 [file Data_Sheet_1.zip › Supplementary Table S2.docx]

**Supplementary Table S2.** Leading publication sources in the Web of Science, Scopus, and PubMed datasets, 2002–2025.

| **Publication source** | **Web of Science** | |  | **Scopus** | |  | **PubMed** | |  | **Leading source in ≥2 datasets** | **Overlap pattern** |
| --- | --- | --- | --- | --- | --- | --- | --- | --- | --- | --- | --- |
|  | **Records, n** | **Rank** |  | **Records, n** | **Rank** |  | **Records, n** | **Rank** |  |  |  |
| *PLoS ONE* | 12 | 1 |  | 10 | 1 |  | 14 | 6 |  | Yes | WoS–Scopus–PubMed |
| *Scientific Reports* | 12 | 1 |  | 8 | 3 |  | 17 | 3 |  | Yes | WoS–Scopus–PubMed |
| *International Journal of Morphology* | 11 | 2 |  | 8 | 3 |  | NT5 | NT5 |  | Yes | WoS–Scopus |
| *Frontiers in Nutrition* | 10 | 3 |  | 9 | 2 |  | 10 | 9 |  | Yes | WoS–Scopus–PubMed |
| *International Journal of Environmental Research and Public Health* | 10 | 3 |  | 9 | 2 |  | 16 | 4 |  | Yes | WoS–Scopus–PubMed |
| *Medicine* | 10 | 3 |  | 7 | 4 |  | 10 | 9 |  | Yes | WoS–Scopus–PubMed |
| *Nutrients* | 9 | 4 |  | 5 | 6 |  | 9 | 10 |  | Yes | WoS–Scopus–PubMed |
| *European Journal of Clinical Nutrition* | 7 | 5 |  | 4 | 7 |  | 7 | 12 |  | Yes | WoS–Scopus–PubMed |
| *Frontiers in Endocrinology* | 7 | 5 |  | 6 | 5 |  | 7 | 12 |  | Yes | WoS–Scopus–PubMed |
| *Frontiers in Physiology* | 7 | 5 |  | 5 | 6 |  | 7 | 12 |  | Yes | WoS–Scopus–PubMed |
| *Sensors* | 7 | 5 |  | 6 | 5 |  | 18 | 2 |  | Yes | WoS–Scopus–PubMed |
| *Blood Pressure Monitoring* | 7 | 5 |  | 6 | 5 |  | 7 | 12 |  | Yes | WoS–Scopus–PubMed |
| *Annals of the National Institute of Hygiene* | NT5 | NT5 |  | 9 | 2 |  | 13 | 7 |  | Yes | Scopus–PubMed |
| *Journal of Physical Education and Sport* | NT5 | NT5 |  | 7 | 4 |  | NT5 | NT5 |  | No | Scopus only |
| *Annual International Conference of the IEEE Engineering in Medicine and Biology Society* | NT5 | NT5 |  | NT5 | NT5 |  | 20 | 1 |  | No | PubMed only |
| *Journal of Artificial Organs* | NT5 | NT5 |  | NT5 | NT5 |  | 15 | 5 |  | No | PubMed only |

NT5, not among the top five source ranks in that database; WoS, Web of Science.

Sources were ranked by publication count within each database. Dense ranking was used, and all sources tied within the top five ranks were retained. Rows include sources ranked within the top five in at least one database. Where available, ranks outside the top five are shown to indicate cross-database presence.
