# Supplementary material for: InBody bioelectrical impedance research in clinical nutrition: a bibliometric analysis of trends, applications, and evidence gaps, 2002–2025
Source: Front Nutr. 2026 Sep 28;13:1896369. doi: 10.3389/fnut.2026.1896369 (PMC13656847; doi:10.3389/fnut.2026.1896369)
Supplement: Supplementary file 1 [file Data_Sheet_1.zip › Supplementary Table S1.docx]

**Supplementary Table S1.** Biblioshiny-derived leading countries/regions based on corresponding author-affiliation metadata in the Web of Science, Scopus, and PubMed datasets, 2002–2025.

| **Countries/regions** | **Web of Science** | |  | **Scopus** | |  | **PubMed** | |  | **Database overlap** |
| --- | --- | --- | --- | --- | --- | --- | --- | --- | --- | --- |
|  | **n** | **Rank** |  | **n** | **Rank** |  | **n** | **Rank** |  |  |
| China | 65 | 1 |  | 55 | 1 |  | 67 | 3 |  | WoS–Scopus–PubMed |
| South Korea | 61 | 2 |  | 50 | 2 |  | 69 | 2 |  | WoS–Scopus–PubMed |
| United States | 37 | 3 |  | 32 | 4 |  | 44 | 4 |  | WoS–Scopus–PubMed |
| Japan | 36 | 4 |  | 36 | 3 |  | 105 | 1 |  | WoS–Scopus–PubMed |
| Poland | 18 | 5 |  | 29 | 5 |  | 19 | 6 |  | WoS–Scopus–PubMed |
| Czech Republic | 14 | 6 |  | 25 | 6 |  | 10 | 10 |  | WoS–Scopus–PubMed |
| Iran | 14 | 6 |  | 12 | 8 |  | 20 | 5 |  | WoS–Scopus–PubMed |
| Brazil | 13 | 7 |  | 10 | 9 |  | 12 | 9 |  | WoS–Scopus–PubMed |
| Hungary | 11 | 8 |  | 8 | 10 |  | 13 | 8 |  | WoS–Scopus–PubMed |
| Slovakia | 11 | 8 |  | 25 | 6 |  | OT10 | — |  | WoS–Scopus |
| Serbia | 9 | 9 |  | 15 | 7 |  | OT10 | — |  | WoS–Scopus |
| Greece | 8 | 10 |  | OT10 | — |  | OT10 | — |  | WoS only |
| Australia | OT10 | — |  | OT10 | — |  | 14 | 7 |  | PubMed only |
| Portugal | OT10 | — |  | OT10 | — |  | 14 | 7 |  | PubMed only |

WoS, Web of Science; OT10, outside the top-10 ranks.

n refers to article counts from the database-specific Biblioshiny country/region output based on corresponding author-affiliation metadata. Ranks were assigned within each database using a shared-rank system; countries/regions with the same article count share the same rank. Countries/regions were included if they appeared within the top 10 ranks in at least one database. OT10 indicates that the country/region was outside the top 10 ranks for that database. Percentages are omitted because the Web of Science, Scopus, and PubMed datasets have different denominators. Because database metadata structures differ, especially for PubMed, these outputs should be interpreted as descriptive affiliation-based indicators rather than standardized corresponding-author country/region counts. No post hoc geopolitical harmonization was applied.
